# From Culture to Sequencing: Evolving Strategies for the Diagnosis of Pediatric Spondylodiscitis

**DOI:** 10.1111/os.70283

**Published:** 2026-03-17

**Authors:** Viola Sbampato, Ahmer Ahmad Khan, Andreas Tsoupras, Giacomo De Marco, Dimitri Ceroni

**Affiliations:** ^1^ Paediatric Orthopedics and Traumatology Unit Geneva University Hospitals and University of Geneva Geneva Switzerland

**Keywords:** diagnosis, narrative review, pediatric, spine, spondylodiscitis

## Abstract

Pediatric spondylodiscitis is a rare but clinically significant infection affecting the intervertebral disc and adjacent vertebral bodies. Diagnostic delays are common due to its nonspecific presentation and the limited sensitivity of conventional microbiological methods. Early and accurate pathogen identification is essential to guide antimicrobial therapy, minimize unnecessary invasive procedures, and prevent long‐term sequelae. Traditional diagnostic tools—including laboratory tests, imaging, blood cultures, biopsy, and histopathological evaluation—remain fundamental but are often insufficient, as they may yield nonspecific results or culture‐negative cases, particularly after prior antibiotic exposure or infection with fastidious organisms. In recent years, molecular approaches, ranging from polymerase chain reaction assays to metagenomic next‐generation sequencing, have markedly improved diagnostic accuracy. These techniques allow rapid and comprehensive pathogen detection, including atypical or previously uncultivable organisms, thereby overcoming many limitations of conventional methods. This narrative review synthesizes current evidence on pediatric spondylodiscitis, outlining its epidemiology, clinical features, and the evolving spectrum of diagnostic strategies—from conventional methods to advanced molecular and sequencing‐based technologies—while discussing future directions in this challenging field.

## Graphical abstract

Diagnostic approaches to pediatric spondylodiscitis have evolved from culture‐based methods with limited sensitivity to advanced molecular and sequencing technologies. Metagenomic next‐generation sequencing (mNGS) enables rapid, culture‐independent pathogen identification, improving diagnostic accuracy, guiding therapy, and reducing invasive procedures in children with this rare spinal infection.

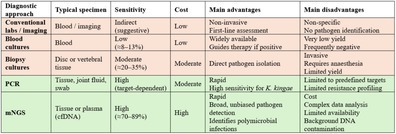

**Abbreviations**
cfDNA: cell‐free DNA
CRP: C‐reactive protein
CT: computed tomography
ESR: erythrocyte sedimentation rate
FDG‐PET: fluorodeoxyglucose positron emission tomography

*K. kingae*

:
*Kingella kingae*

mNGS: metagenomic next‐generation sequencing
MRI: magnetic resonance imaging
NAAAs: nucleic acid amplification assays
PCR: polymerase chain reaction
PSD: pediatric spondylodiscitis
qPCR: quantitative real‐time polymerase chain reaction
rRNA: ribosomal ribonucleic acid
WBC: white blood cell count

## 1 Introduction

Pediatric spondylodiscitis (PSD) is a rare but clinically significant infection involving the intervertebral disc and adjacent vertebral bodies. Although less common than in adults, it remains an important cause of morbidity and healthcare utilization, requiring close collaboration among pediatricians, orthopedists, infectious disease specialists, and radiologists [1, 2]. The incidence is approximately 1 in 250,000, accounting for about 2%–4% of infectious bone diseases, with a peak incidence in children younger than 4 years [3, 4, 5].

Theoretically, the infection arises either through hematogenous spread, direct external inoculation (such as following trauma, surgery, puncture, or epidural procedures), or contiguous spread from adjacent tissues [6]. However, in children, hematogenous dissemination represents the almost exclusive route of infection, with most cases of PSD arising from a primary focus [7, 8]. The higher incidence observed in early childhood is largely explained by the unique vascular anatomy characteristic of this age group, in which trans‐apophyseal vessels connect adjacent vertebral bodies through the disc space, facilitating bacterial seeding of the disc–vertebral complex. These vascular channels progressively regress after approximately 8 years of age [9, 10, 11, 12].

Clinical manifestations are often vague and initially attributed to more common conditions, making diagnosis challenging and frequently delayed—sometimes for weeks or months [12, 13, 14, 15, 16]. This delay is clinically significant, as prompt recognition is essential to prevent potential serious complications such as vertebral collapse, spinal deformity, neurologic impairment, or chronic pain [1, 2, 6, 11]. Initial evaluation therefore relies on laboratory investigations and imaging, although these are often nonspecific, particularly in early disease [1, 2, 3, 6, 8, 11, 15, 16, 17, 18, 19, 20, 21, 22, 23, 24, 25, 26, 27, 28, 29]. When noninvasive methods fail to establish a bacterial diagnosis, invasive procedures such as biopsy or aspiration may be undertaken. The obtained sample allows histopathological analysis to differentiate between neoplastic and inflammatory processes, while microbiological cultures can be performed to identify a causative pathogen [30, 31, 32, 33, 34].

In recent years, molecular diagnostic techniques such as nucleic acid amplification assays (NAAAs) have emerged, transforming the landscape of infectious disease detection [35, 36, 37, 38]. This spectrum of technologies—from the various types of polymerase chain reaction (PCR) to metagenomic next‐generation sequencing (mNGS)—has collectively shifted the diagnostic paradigm in pediatric spinal infections [38, 39, 40, 41, 42].

This narrative review synthesizes evidence on PSD, focusing on the evolution from conventional culture‐based diagnostic methods to sequencing‐driven approaches, particularly mNGS. It is based on a comprehensive, non‐systematic literature search conducted in PubMed and Embase, including articles published in English up to October 2025. Search terms comprised combinations of *pediatric spondylodiscitis*, *discitis*, *spinal infection*, *polymerase chain reaction*, *molecular diagnostics*, and *metagenomic next‐generation sequencing*.

## 2 Epidemiology, Microbiological Spectrum, and Clinical Presentation

PSD is an uncommon infection with a poorly defined true incidence, largely due to variability in diagnostic criteria, imaging availability, and clinical awareness. Nevertheless, the widespread adoption of MRI has led to increased detection in recent years, with studies estimating that PSD accounts for approximately 3%–7% of pediatric osteoarticular infections requiring hospitalization and an annual incidence of two to five cases per million children [25, 43, 44].

According to recent studies, PSD can be divided into three distinct age‐related clinical forms, each with characteristic microbiological profiles [45]. The neonatal form, affecting infants younger than 6 months, is the rarest but also the most severe presentation, often associated with bacteremia, sepsis, or multiple infectious foci [45, 46]. During this early period, passively transferred maternal immunoglobulins provide partial protection against certain pathogens, with *Staphylococcus aureus* remaining the predominant organism, accounting for up to 80% of cases [45]. The infantile form, occurring between 6 and 48 months of age, accounts for 60%–80% of cases and corresponds to the developmental window in which maternally acquired antibodies have waned while the child's humoral immunity is still maturing [45]. During this stage, frequent upper respiratory infections and persistent trans‐apophyseal vascular channels may facilitate bacterial dissemination to the disc–vertebral complex [6, 11, 14, 45]. In this age group, *Kingella kingae* ( *K. kingae* ) is the principal pathogen, identified in up to 80%–90% of microbiologically confirmed cases [35, 47]. After the age of 4 years, the childhood–adolescent form becomes more prevalent, characterized by a shift back to *S. aureus* as the predominant causative organism, often accompanied by more systemic symptoms such as fever and malaise [14, 35, 45].

Sex distribution is roughly balanced, though several multicenter cohorts have noted a slight male predominance (55%–65%) [6, 13, 48]. Overall, sex does not appear to be a major determinant of disease susceptibility, as other studies show no significant difference [10, 14, 49]. The lumbar spine is the most frequently involved region, accounting for more than half of all cases [8, 12, 13, 50, 51, 52]. Within this region, the L4–L5 level is most often affected, followed by L5–S1 and L3–L4 [13, 48, 53, 54]. Thoracic and cervical infections are less frequent, and multivertebral involvement, though uncommon at presentation, may develop when diagnosis is delayed, especially in tuberculous forms [6, 55, 56].

The clinical manifestations of PSD are typically subtle and nonspecific, often resulting in diagnostic delays of several weeks and, in some cases, up to a year [10, 14, 27]. In infants and toddlers, presentation may be limited to irritability, reduced appetite, or refusal to sit, stand, crawl, or walk [1, 6, 13, 14, 18, 44, 48, 49, 57]. Parents may also report crying or distress when the child is lifted under the arms or during diaper changes, reflecting spinal discomfort [58]. In older children and adolescents, the condition usually presents with localized back pain that worsens with movement and at night, accompanied by stiffness of the back and neck or a limp [1, 13, 16, 48, 59, 60, 61]. Case series and reviews observe that systemic symptoms such as low‐grade fever or preceding viral illness are present in fewer than half of cases (15%–30%) [2, 6, 18, 59]. Neurological complications are uncommon and typically reflect advanced disease, including spinal cord or nerve root compression, epidural extension, abscess formation, or vertebral collapse [3, 6, 10, 11, 16, 26, 62, 63, 64, 65].

## 3 Conventional Diagnostic Approaches

The diagnostic workup of PSD traditionally relies on a combination of laboratory tests, microbiological investigations, and imaging studies. Each modality contributes valuable information, yet none is sufficient on its own, and their individual limitations account for the high frequency of diagnostic delays in children.

### 3.1 Laboratory Investigations

Laboratory evaluation is usually the first step in a child with suspected PSD [8]. White blood cell count (WBC), erythrocyte sedimentation rate (ESR), and C‐reactive protein (CRP) are the most frequently assessed inflammatory markers. These parameters, however, must be interpreted cautiously, as their values vary with patient age, infecting organism, and disease chronicity [8, 13, 17, 59, 61, 66, 67]. Across large multicenter studies, ESR is elevated in most cases—often exceeding 40 mm/h in more than 80% of patients—whereas CRP tends to show only moderate elevation and may even remain normal, especially in chronic infections [11, 12, 13, 16, 66, 67, 68]. Accordingly, ESR is generally considered more sensitive for initial diagnosis, while CRP is more useful for monitoring disease activity and therapeutic response [69]. Leucocytosis is relatively uncommon in pediatric spondylodiscitis, with white blood cell counts exceeding 12,000/mm^3^ in only about one‐third of cases, typically in acute or subacute presentations [11, 13, 59, 66, 67]. As most children present with normal or only mildly elevated WBC counts, this diagnostic marker has limited sensitivity [10, 11, 14, 49, 59]. Thrombocytosis, however, is reported in 50%–60% of cases, especially among toddlers and preschool‐aged children, but neither sensitive nor specific enough to serve as standalone diagnostic criteria [12, 13, 66].

### 3.2 Imaging

Imaging plays a central role in the evaluation of PSD, both for early detection and for monitoring progression.

#### 3.2.1 Plain Radiographs

Plain radiographs are generally obtained as the initial imaging modality when PSD is suspected; however, they lack both sensitivity and specificity for early detection [2, 11]. Early images are frequently normal or show only subtle disc space narrowing, with radiographic abnormalities typically becoming apparent several weeks after symptom onset and in only about 50% of cases [10, 11, 18]. Late findings reflect permanent structural sequelae, including segmental collapse, vertebral fusion, and spinal deformities such as kyphosis or scoliosis [2, 6, 10, 11, 17, 18, 21, 27, 28, 68].

#### 3.2.2 Computed Tomography

Computed tomography (CT) is now rarely used for the primary diagnosis of PSD. Despite providing excellent anatomic detail, its limited sensitivity and exposure to ionizing radiation restrict its use in children [3, 8]. CT is mainly reserved for preoperative planning or for guiding percutaneous biopsy or aspiration procedures [63].

#### 3.2.3 Magnetic Resonance Imaging

Magnetic resonance imaging (MRI) has become the gold standard for diagnosing PSD, with a reported sensitivity between 85% and 94% and specificity around 66%–93% [70]. MRI sensitivity increases over time, identifying characteristic changes in approximately 58% of cases within the first 2 weeks of symptom onset and up to 82% thereafter [29]. This noninvasive modality allows early and detailed visualization of pathological changes such as bone marrow oedema, loss of disc height, endplate irregularity, and soft‐tissue or marrow alterations including vascularized fibrous tissue, fatty transformation, subchondral fibrosis, and sclerosis [15, 58, 63, 71]. Typical signal changes include low T1‐weighted and high T2‐weighted signal intensity in affected vertebral bodies [13]. Gadolinium enhancement improves delineation of infected endplates, helps differentiate phlegmon from abscess, and defines the extent of disease and associated complications such as spinal cord compression [63, 72, 73, 74, 75]. Despite its accuracy, MRI remains limited by the need for sedation in young children as cooperation is often limited in this age group [76, 77].

#### 3.2.4 Nuclear Medicine

Nuclear medicine techniques such as bone scintigraphy, gallium‐67, or FDG‐PET scans are now rarely used for pediatric spondylodiscitis, being largely replaced by MRI and retained only in selected centers or countries where MRI is unavailable [78].

### 3.3 Bacteriological Diagnostic Approaches

#### 3.3.1 Blood Cultures

Blood cultures remain a cornerstone of conventional practice because a positive result can guide therapy and obviate the need for invasive procedures. However, their diagnostic yield in pediatric PSD is disappointingly low. Across multiple series, blood cultures were positive in only 8%–13% of children, far below the rates observed in other pediatric osteoarticular infections [6, 14, 18, 79].

#### 3.3.2 Cultures of Biopsy or Aspiration Material

Percutaneous biopsy or aspiration (either open or CT‐guided) of the affected disc or vertebral body is performed in children only in case of diagnostic uncertainty, while all other invasive techniques have failed, after there is a failure to respond to antibiotics, or if unusual pathogens are suspected [6, 63, 80]. Thus, while it represents theoretically the best chance at pathogen recovery, biopsy is not a routine part of pediatric practice due to the risks associated with anesthesia and needle access to the spine. In addition, even when performed, the diagnostic yield is modest as culture positivity ranges from 20% to 35% in most series, and negative results remain common [6, 49, 80, 81].

#### 3.3.3 Histopathological Assessment

Once a sample is obtained, microscopic examination can help distinguish between different types of infections. Pyogenic spondylodiscitis typically shows acute neutrophilic infiltration with extensive necrosis, whereas tuberculous infection is characterized by granulomatous inflammation and caseous necrosis [30, 31, 32, 33, 34]. In studies of primary spinal infections, histopathological analysis demonstrated a sensitivity of 81%–91% and a specificity of 100%, supporting its value as a complementary tool alongside microbiological investigations [30, 31, 82]. In our latitudes, the use of histopathological assessment remains rather confidential since the incidence of mycobacterial spondylitis is currently very low.

## 4 Molecular and Novel Diagnostic Strategies

Over the past 30 years, nucleic acid amplification assays (NAAAs) have emerged as transformative tools in the diagnostic approach to pediatric spinal infections [42, 83, 84, 85, 86]. By detecting microbial DNA or RNA independent of culture, they provide sensitive, rapid, and culture‐independent pathogen identification [37, 87, 88]. NAAAs encompass a spectrum of technologies—from broad‐range and targeted polymerase chain reactions (PCR) to metagenomic next‐generation sequencing (mNGS)—and together they bridge the gap left by conventional methods.

Broad‐range PCR assays were among the first molecular tools introduced into clinical use for diagnosing osteoarticular infections. They amplify genetic sequences from a wide variety of microorganisms using primers that bind to highly conserved regions of genes, such as the bacterial 16S rRNA gene. Compared with conventional culture, broad‐range PCR has demonstrated higher bacterial detection rates, identifying organisms in 42%–60% of cases in which cultures succeeded in only 28.9%–50% [89, 90, 91]. Reported diagnostic performance varies across studies, with sensitivity ranging from 75% to 88.5% and specificity from 71% to 100%, depending on laboratory protocols and contamination control measures [90, 92, 93, 94]. However, species‐level identification usually requires sequencing of the amplified fragment, and interpretation may be limited by environmental contamination, high background human DNA, or difficulty resolving polymicrobial infections [95, 96, 97].

Targeted PCR was later introduced, enabling rapid and sensitive detection of predefined organisms by amplifying sequences specific to known pathogens, even in samples with a low bacterial load or after prior antibiotic exposure [98, 99]. Its clinical impact has been most evident in toddlers between 6 months and 4 years of age, in whom targeted PCR has identified *K. kingae* as the leading cause of pediatric spondylodiscitis, with reported detection rates of up to 87.5%–100%, thereby substantially improving microbiological confirmation [12, 13, 66, 67]. However, its main limitation is that it cannot detect unexpected or atypical pathogens, as only those included in the assay panel will be identified [100, 101].

Real‐time PCR (qPCR) was subsequently introduced as an evolution of targeted PCR, enabling quantification of microbial DNA in real time using fluorescent probes [102]. Compared with conventional PCR, it offers reduced contamination risk due to its closed‐tube format, faster turnaround times, and higher sensitivity, with reported values of 85–100% in osteoarticular infections depending on the pathogen and specimen type [100, 103, 104, 105]. *K. kingae* ‐specific real‐time PCR assays have consistently demonstrated superior sensitivity compared with broad‐range 16S rRNA gene PCR, significantly increasing detection rates in joint fluid, especially in culture‐negative cases [43, 106, 107, 108]. Nonetheless, its diagnostic scope remains limited to predefined organisms explicitly included in the assay design [103], restricting its utility in atypical or polymicrobial infections.

Multiplex PCR, introduced in the early 21st century, builds upon conventional PCR by enabling the simultaneous detection of multiple bacterial or viral targets within a single reaction [109, 110]. This next step in molecular diagnostic techniques reduces turnaround time and allows identification of coinfections or unexpected pathogens [111, 112, 113, 114]. In pilot pediatric osteoarticular studies, customized multiplex panels achieved an overall diagnostic yield of approximately 91%, outperforming single‐target assays in selected cohorts when used in combination with standard culture methods [115]. However, its use in pediatric septic disease remains limited, as most commercially available panels are designed for respiratory, cerebrospinal, or bloodstream infections rather than osteoarticular infections. Nevertheless, customized multiplex assays targeting key pediatric osteoarticular pathogens—including *K. kingae* , *S. aureus* , and *Streptococcus pneumoniae* —have shown encouraging results in pilot studies but are not yet widely available [101, 116].

While PCR‐based methods rely on predefined targets, a more comprehensive and transformative advance among NAAAs emerged at the end of the first decade of the 21st century with the introduction of metagenomic next‐generation sequencing (mNGS). In contrast to conventional PCR‐based methods, mNGS analyses all nucleic acids present in a specimen and aligns them against extensive genomic databases [117]. This unbiased, hypothesis‐free approach enables simultaneous detection of bacteria, viruses, fungi, and parasites in a single assay [117].

The method is both rapid and wide‐ranging—capable of identifying more than 1400 microbial species within 1–2 days [118]. In spinal infections, mNGS has consistently demonstrated higher sensitivity than conventional culture, with reported detection rates in recent literature between 70% and 89%, even in children who have received antibiotics before sampling [42, 86, 119]. These features make it particularly valuable when the infectious etiology is unclear, such as in immunocompromised hosts or cases involving rare or unexpected organisms [42, 120, 121, 122, 123]. Furthermore, mNGS can readily identify fastidious or slow‐growing bacteria—including *Brucella* species, *Mycobacterium tuberculosis* , and uncommon fungi—that are notoriously difficult to cultivate, thereby accelerating diagnosis and clinical decision‐making [118, 122, 123, 124].

Beyond its broad pathogen detection capabilities, mNGS offers several analytical advantages that enhance clinical utility. The technique can detect mixed infections and quantify the relative abundance of each organism, which is particularly useful in polymicrobial or chronic cases [123, 125, 126, 127]. Recent advances also enable integration of host‐response profiling, where transcriptomic and metabolomic signatures are analyzed to help distinguish between infection and colonization or to gauge disease severity. Importantly, mNGS can simultaneously identify virulence determinants and antimicrobial resistance genes, supporting more precise therapeutic strategies and facilitating early optimization of antibiotic regimens [121, 122, 123, 128, 129, 130, 131].

Despite these advantages, the implementation of mNGS in clinical practice remains constrained by several important limitations. Data interpretation can be challenging, as sequencing frequently detects background DNA from environmental contaminants or commensal flora, which may obscure true pathogens [120, 122, 132, 133]. High levels of host‐derived DNA can further reduce sensitivity, especially in infections with low microbial burden. Additionally, the method is expensive, technologically demanding, and reliant on sophisticated bioinformatics pipelines that lack cross‐laboratory standardization [99, 117, 118, 120, 121, 133, 134]. Until now, no mNGS assay has yet obtained FDA approval, and discrepancies in sample processing, sequencing platforms, and analytical methods hinder reproducibility and inter‐study comparability [133]. Continuous curation and expansion of genomic reference databases are also essential to maintain diagnostic accuracy and ensure reliable detection of emerging or rare pathogens [117]. Despite these challenges, mNGS represents a powerful and rapidly evolving technology that is likely to become an integral component of future diagnostic algorithms for pediatric spinal infections.

## 5 Indirect Diagnosis of PSD

Indirect diagnostic strategies for PSD have been developed over the past decade to improve pathogen detection, reduce costs, and minimize the need for invasive spinal sampling, which may be contraindicated or technically challenging (Table 1).

**TABLE 1:** Summarizes the main diagnostic approaches for PSD, comparing typical specimens, diagnostic yield, costs, and principal advantages and limitations.

Diagnostic approach	Sample/modality	Sensitivity	Cost	Main advantages	Main disadvantages
Conventional labs/imaging	Blood/imaging	Indirect (suggestive)	Low	Noninvasive First‐line assessment	Nonspecific No pathogen identification
Blood cultures	Blood	Low (~8%–13%)	Low	Widely available Guides therapy if positive	Very low yield Frequently negative
Biopsy cultures	Disc or vertebral tissue	Moderate (~20%–35%)	Moderate	Direct pathogen isolation	Invasive Requires anesthesia Limited yield
PCR	Tissue, joint fluid, swab	High (target‐dependent)	Moderate	Rapid High sensitivity for *K. kingae*	Limited to predefined targets Limited resistance profiling
mNGS	Tissue or plasma (cfDNA)	High (~70%–89%)	High	Rapid Broad, unbiased pathogen detection Identifies polymicrobial infections	Cost Complex data analysis Limited availability Background DNA contamination

Metagenomic NGS has also been adapted into a “liquid biopsy” format that analyses microbial cell‐free DNA (cfDNA) in plasma to detect pathogens circulating in the bloodstream and causing focal infections. This noninvasive approach is particularly promising in children, where invasive spinal sampling is often avoided, and it reduces the need for costly specimen collection [135]. In a recent multicenter study, plasma mNGS successfully identified *K. kingae* in all cases of PSD that had previously yielded negative blood cultures [135].

Another well‐established indirect diagnostic method for PSD is the detection of *K. kingae* DNA in the oropharynx. Colonization of the upper respiratory tract often precedes hematogenous dissemination to bones or intervertebral discs. Consequently, real‐time PCR assays targeting the *rtxA* or *mdh* genes on oropharyngeal swabs have demonstrated very high diagnostic performance in children with confirmed osteoarticular infections, including PSD [66, 103, 104, 105, 106, 107, 136].

## 6 Summary and Proposed Diagnostic Pathway for PSD

**FIGURE 1:**
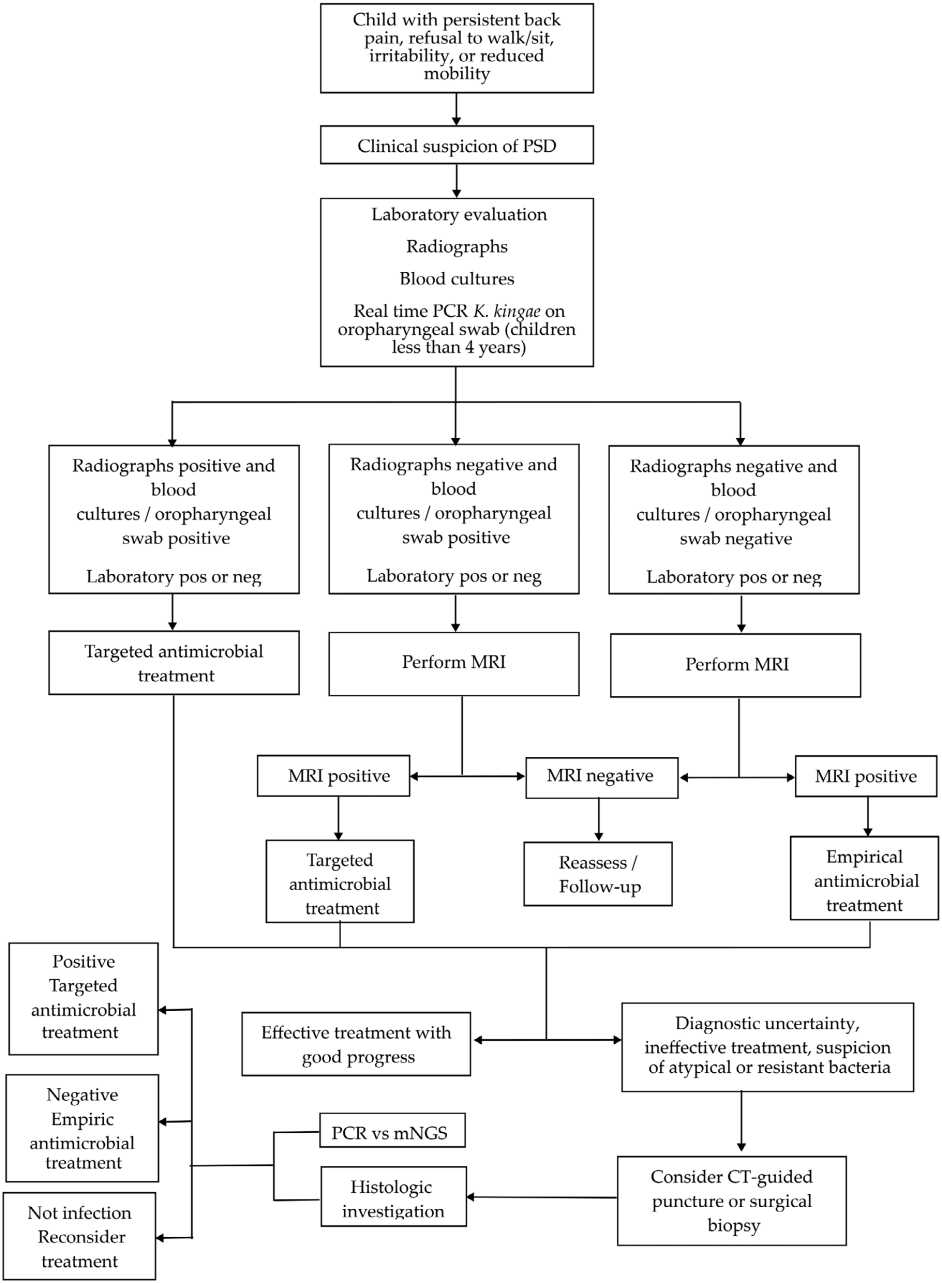
Flowchart illustrating a stepwise diagnostic pathway for pediatric spondylodiscitis, integrating clinical suspicion, laboratory evaluation, imaging, and microbiological and molecular investigations to guide targeted antimicrobial therapy.

Based on the diagnostic approaches discussed above, a pragmatic stepwise diagnostic pathway for PSD is proposed, aiming to balance early diagnosis with avoidance of unnecessary invasive or resource‐intensive investigations (Figure 1).

### 6.1 Step 1—Clinical Suspicion

PSD should be suspected in children presenting with persistent back pain, refusal to walk or sit, unexplained irritability, or reduced mobility, often in the absence of marked systemic symptoms.

### 6.2 Step 2—Initial Imaging: Conventional Radiography

Conventional spinal radiographs should represent the first‐line imaging modality in the primary evaluation of suspected pediatric spondylodiscitis. Although radiographs are relatively insensitive in the early stages of disease, they play a key role in excluding alternative non‐infectious causes of symptoms, such as trauma, spinal deformities, or neoplastic lesions. When radiographs identify a non‐infectious cause explaining the clinical presentation, further infectious investigations, including laboratory tests, blood cultures, and oropharyngeal swabs, are not pursued. Conversely, when radiographs are normal or show findings suggestive of spondylodiscitis, additional diagnostic evaluation is warranted.

### 6.3 Step 3—Laboratory and Microbiological Evaluation

In children with negative or suggestive radiographs, baseline laboratory investigations should be performed, including ESR, CRP, WBC count, and platelet count. Inflammatory markers provide supportive but nonspecific evidence of infection; ESR and platelet count are often abnormal in PSD, particularly in younger children.

Blood cultures should be obtained before antibiotic initiation whenever feasible, despite their limited diagnostic yield.

In children younger than 4 years, a real‐time PCR for *K. kingae* on an oropharyngeal swab should be systematically considered.

### 6.4 Step 4—Selection for MRI

Spinal MRI should be planned in children with radiographs suggestive of spondylodiscitis, abnormal laboratory findings such as elevated ESR and/or thrombocytosis, regardless of blood culture results, or in those with positive blood cultures or a positive *K. kingae* oropharyngeal PCR, particularly in children aged 6–48 months. In contrast, in children younger than 4 years with normal radiographs, normal ESR and platelet count, and a negative *K. kingae* oropharyngeal PCR, a strategy of close clinical surveillance without immediate MRI may be appropriate.

### 6.5 Step 5—Integration of MRI and Microbiological Results

MRI findings, together with blood culture and oropharyngeal PCR results, should guide further diagnostic and therapeutic decision‐making, including initiation of targeted or empirical antimicrobial therapy.

### 6.6 Step 6—Advanced Diagnostics and Invasive Sampling (Selected Cases)

In cases of diagnostic uncertainty, poor clinical response to empirical therapy, or suspicion of atypical or resistant pathogens, additional investigations should be considered. These include:

- Targeted PCR assays and metagenomic next‐generation sequencing (mNGS) on tissue samples.
- Plasma‐based mNGS (cell‐free DNA) when invasive sampling is not feasible.

Percutaneous biopsy or aspiration should be reserved for selected cases, particularly when tuberculosis or fungal infection is suspected or when molecular diagnostics are unavailable. Histopathological analysis should complement microbiological testing whenever tissue is obtained.

## 7 Study Limitations and Future Perspectives

This narrative review has inherent limitations related to the rarity of PSD and the heterogeneity of the available literature, which is largely based on retrospective studies and small cohorts. In addition, while mNGS represents a major advance in pathogen detection, its routine clinical implementation remains constrained by high costs, limited availability, lack of standardized laboratory and bioinformatics workflows, and challenges in result interpretation, particularly in distinguishing true pathogens from background DNA contamination. The need for specialized expertise further restricts its use to selected centers. Future efforts should focus on assay standardization, improved contamination control, validated analytical pipelines, and cost reduction through technological advances and broader adoption. Multicenter prospective studies and international collaborations will be essential to define the optimal role of mNGS within diagnostic algorithms for pediatric spinal infections.

## 8 Conclusion

PSD remains a rare but clinically significant condition, distinguished by its subtle onset, diagnostic delays, and potential for long‐term sequelae. Although inflammatory markers, imaging, and blood cultures remain standard diagnostic tools, their limitations contribute to delayed diagnosis and a high proportion of culture‐negative cases.

The emergence of NAAAs, particularly mNGS, marks a paradigm shift in the diagnostic landscape, and by integrating such tools into existing diagnostic pathways, clinicians can achieve earlier, more precise diagnoses and improved treatment efficacy. Looking forward, advances in molecular diagnostics, together with the establishment of international registries and multicenter collaborations, offer the potential for a more standardized and evidence‐based approach to PSD. In summary, PSD exemplifies the challenges of managing rare pediatric infections in an evolving technological era. By combining traditional clinical vigilance with novel molecular tools, clinicians will achieve earlier diagnoses, more individualized therapies, and ultimately, improved short‐ and long‐term outcomes, both physical and mental, for affected children worldwide.

## Author Contributions

Viola Sbampato conceptualized the study, drafted the initial manuscript, and critically reviewed and revised the manuscript. Ahmer Khan, Andreas Tsoupras, and Giacomo De Marco reviewed and critically revised the manuscript and approved the final version. Dimitri Ceroni conceptualized the study, coordinated and supervised the redaction, and critically reviewed and revised the manuscript. All authors approved the final manuscript as submitted and agree to be accountable for all aspects of the work.

## Funding

The authors have nothing to report.

## Disclosure

All claims expressed in this article are solely those of the authors and do not necessarily represent those of their affiliated organizations, or those of the publisher, the editors, and the reviewers. Any product that may be evaluated in this article or claim that may be made by its manufacturer is not guaranteed or endorsed by the publisher.

## Ethics Statement

The authors have nothing to report.

## Conflicts of Interest

The authors declare no conflicts of interest.

## Data Availability

Data sharing not applicable to this article as no datasets were generated or analyzed during the current study.
